# A rare case of seronegative neurobrucellosis

**DOI:** 10.4103/0256-4947.67084

**Published:** 2010

**Authors:** Suda Tekin-Koruk, Fazilet Duygu, Bensu Gursoy, Leman Karaagac, Mehmet Bayraktar

**Affiliations:** From Harran University, Infectious Diseases and Clinical Microbiology Yenisehir Campus, Sanliurfa, Turkey

## Abstract

Neurobrucellosis is one of the complications of brucellosis. We report a rare case of a 17-year-old girl with seronegative neurobrucellosis and depression and diplopia. Results of agglutination tests for *Brucella* both in serum and CSF were negative. Diagnosis was made only by positive culture of *Brucella* mellitensis with inoculation of the patient’s cerebrospinal fluid in a BACTEC 9050 System. The patient was successfully treated using ceftriaxone, doxycycline and rifampicin therapy for six months.

Human brucellosis is a multisystem zoonotic disease with varied manifestations. It is endemic in many parts of the world, including the Middle East, the Arabian Peninsula, South Asia and Turkey.^1^,^2^ In 2004, the incidence of brucellosis in Turkey was 25.67/100000 population.^3^ Neurologic manifestations of brucellosis occur in 3% to 5% of patients.^4^,^5^ The symptoms of neurobrucellosis includes meningitis, encephalitis, myelitis, radiculoneuronitis, brain abscess, epidural abscess, granuloma, and demyelinating and meningovascular syndromes.^6^ The disease is diagnosed by positive serology (Rose Bengal agglutination test and standard tube dilution test) with neurological dysfunction not explained by other neurologic diseases or isolation of other bacteria either in cerebrospinal fluid (CSF) or blood concomitant with abnormal CSF findings (increase in leucocytes and protein, decrease in glucose levels).^7^,^8^ We present a rare case of seronegative neurobrucellosis with depression and diplopia caused by *Brucella* melitensis and subsequent treatment by the combined therapy in this report.

## CASE

A 17-year-old girl presented with diplopia, headache, nausea and vomiting of 6 weeks’ duration with no fever or sweating. Headache was continuous and nonpulsatile and associated with vomiting, which was nonprojectile in nature. Pain in the lumbosacral region was moderate. There was no history of convulsions, but the patient displayed absurd speech, depression and lack of communication. She had visited the neurology department and had taken several drugs like analgesics and antidepressants, but her complaints worsened.

On physical examination she had a fever of 36.8°C, her pulse was 96/minute and regular, blood pressure 90/60 mm Hg, and the respiratory rate was 20 breaths per minute. A neurological examination showed diplopia. The fundus examination was normal. Her mental status was normal. There was no neck stiffness, Kernig sign or neurologic deficits on physical examination. The cardiovascular system, respiratory system and gastrointestinal system examinations were unremarkable.

Hematological examination revealed a hemoglobin of 12.8 g/dL, white blood cell 5470/mm^3^ (leukocytes included 64% lymphocytes, 24% neutrophils and 12% monocytes), high sensitivity C-reactive protein was 2.7 mg/L, and an erythrocyte sedimentation rate of 40 mm/h. Other investigations, including renal and liver function tests and urine analysis, were within normal limits. Blood serological studies for brucellosis, toxoplasmosis, cytomegalovirus and herpes virus were negative. Neuroimaging studies (cranial CT and MRI) were normal. Nearly six weeks following onset of symptoms, the patient was admitted to the hospital immediately. CSF was taken in our clinic by performing lumbar puncture. The opening pressure was 240 mm H_2_O. Examination of the CSF revealed 430 lymphocytes per mm^3^, with 515 mg/dL protein and 44 mg/dL glucose (serum glucose: 104 mg/dL). No microorganisms were seen on Gram, methylene blue or acid-fast stains of CSF. Simultaneously a 2-mL CSF sample was inoculated into a blood bottle, BACTEC 9050 (Becton, Dickinson and Company, USA).*Brucella melitensis* was isolated on the fifth day of incubation and identified according to the conventional methods. The serum Rose Bengal test was negative. Both CSF *Brucella* agglutination and serum Coombs tests were also negative. After diagnosis of neurobrucellosis, the patient was re-interrogated for specific risk factors for brucellosis. Her family had animals (sheep) and her diet included the consumption of unpasteurized cheese.

She was treated with ceftriaxone (2 g twice daily), doxycycline (200 mg/day) and rifampicin (600 mg/day) orally. The patient recovered completely, her diplopia disappeared, and the CSF leukocyte count decreased to 100 per mm^3^ on the fifth day of therapy. After 3 weeks, she was discharged from the hospital and advised to continue the anti-*Brucella* medication. The CSF analysis was returned to normal within 5 months. The duration of therapy was 6 months on the whole. She was healthy. No relapse was seen 6 months after the end of therapy.

## DISCUSSION

Neurobrucellosis, a form of chronic meningitis, has an onset measured in weeks to months and is generally defined when symptoms, signs, and the CSF remain abnormal for at least 4 weeks. Acute purulent bacterial meningitis is clinically defined as a syndrome characterized by the onset of meningeal symptoms over the course of hours to up to several days. 9 Neurobrucellosis has a diverse clinical presentation. It can affect both the central and peripheral nervous system. The central nervous system is involved in less than 5% of cases.^6^ It is easily confused with many other neurological, neurosurgical, or even psychiatric disorders. Patients may visit psychiatry departments owing to the chronic character of the disease.^10^ Similarly, our case was previously examined by neurologist twice with no response.

Clinical manifestations of neurobrucellosis vary widely. Meningitis is the most frequent one. However, it has been noted that <50% of patients with *Brucella* meningitis exhibit meningeal signs.^4^ We did not encounter any meningeal symptom other than headache and diplopia in our patient. The diagnosis of nuerobrucellosis depends on the demonstration of meningeal inflammation and detection of specific antibodies in the CSF because Gram stains of CSF are usually negative and cultures are positive in less than one quarter of cases. CSF analysis reveals a lymphocytic pleocytosis, elevated protein and low to normal glucose.^6^,^11^ In a large series, 96% of the cases are seropositive, but cases may be seronegative, as in our case.^12^ Our patient’s CSF culture yielded *B melitensis*, but serologic tests for brucellosis were negative both in blood and CSF. Awareness of the existence of brucellosis is essential, especially in endemic regions.

Isolation of the organism from specimens like blood, bone marrow or other tissues makes the diagnosis certain. The rate of isolation ranges from 15% to more than 90% depending on the methods used.^13^ Most laboratories now use an automated blood culture system (e.g., BACTEC). In the BACTEC culture system, the mean detection time for *Brucella* is reported to be 2 to 7 days.^14^ Ozisik et al reported that *Brucella* spp. was identified using BACTEC 9120 blood culture system on the fifth day after inoculation.^15^ In our case, an automated culture system, BACTEC 9050 yielded *Brucella* spp. from the patient’s CSF. BACTEC culture systems can yield the organism faster, resulting in isolation and identification of *Brucella* in patients with an epidemiologically relevant history, who are suspected to have brucellosis.

The treatment of neurobrucellosis poses special problems and there is no single opinion regarding the optimal regimen. Nevertheless, most authorities recommend the use of doxycycline in combination with two or more antimicrobial agents that penetrate the blood-brain barrier well.^5^,^6^ Bodur et al treated their patients successfully with a combination of doxycycline plus rifampin and ceftriaxone in their study.^16^ On the other hand, after isolation of *Brucella* spp. from CSF, Ozısık et al treated their patient with rifampin (600 mg/day orally) and trimethoprimsulfamethoxazole (TMP-SMX) (160 mg twice a day) for five months (doxycycline was not tolerated and was stopped on the fifth day).^15^ Gul et al recommends parenteral ceftriaxone as an initial alternative in the management of neurobrucellosis and the duration of therapy should be a minimum of 6 months with suitable antibiotics.^17^ In our case, we continued our regimen for six months.

This case illustrates that brucellosis can present in various clinical forms in endemic areas and can be characterized by severe complications. It may be difficult to diagnose brucellosis, especially in patients with atypical syndromes. It should be kept in mind, that in endemic areas like Turkey, brucellosis should be suspected in patients who experience unexplainable neurological and psychiatric problems and the CSF should be adequately analyzed.
